# Care during the third stage of labour: A postal survey of UK midwives and obstetricians

**DOI:** 10.1186/1471-2393-10-23

**Published:** 2010-05-21

**Authors:** Diane Farrar, Derek Tuffnell, Rebecca Airey, Lelia Duley

**Affiliations:** 1 Bradford Institute for Health Research, Bradford Royal Infirmary, Bradford, BD9 6RJ, UK; 2 Women's and Newborn Unit, Bradford Royal Infirmary, Bradford, BD9 6RJ, UK; 3 Obstetric Epidemiology Group, Centre for Epidemiology and Biostatistics, University of Leeds, Leeds, LS2 9JT, UK

## Abstract

**Background:**

There are two approaches to care during the third stage of labour: Active management includes three components: administration of a prophylactic uterotonic drug, cord clamping and controlled cord traction. For physiological care, intervention occurs only if there is clinical need. Evidence to guide care during the third stage is limited and there is variation in recommendations which may contribute to differences in practice. This paper describes current UK practice during the third stage of labour.

**Methods:**

A postal survey of 2230 fellows and members of the Royal College of Obstetricians and Gynaecologists (RCOG) and 2400 members of the Royal College of Midwives was undertaken. Respondents were asked about care during the third stage of labour, for vaginal and caesarean births and their views on the need for more evidence to guide care in the third stage. The data were analysed in Excel and presented as descriptive statistics.

**Results:**

1189 (53%) fellows and members of the RCOG and 1702 (71%) midwives responded, of whom 926 (78%) and 1297 (76%) respectively had conducted or supervised births in the last year. 93% (863/926) of obstetricians and 73% (942/1297) of midwives report 'always or usually' using active management. 66% (611/926) of obstetricians and 33% (430/1297) of midwives give the uterotonic drug with delivery of the anterior shoulder; this was intramuscular Syntometrine^® ^for 79% (728/926) and 86% (1118/1293) respectively. For term births, 74% (682/926) of obstetricians and 41% (526/1297) of midwives clamp the cord within 20 seconds, as do 57% (523/926) and 55% (707/1297) for preterm births. Controlled cord traction was used by 94% of both obstetricians and midwives. For caesarean births, intravenous oxytocin was the uterotonic used by 90% (837/926) of obstetricians; 79% (726/926) clamp the cord within 20 seconds for term births as do 63% (576/926) for preterm births.

Physiological management was used 'always or usually' by 2% (21/926) of obstetricians and 9% (121/1297) of midwives. 81% (747/926) of obstetricians and 89% (1151/1297) of midwives thought more evidence from randomised trials was needed; the most popular question was when is best to clamp the cord.

**Conclusions:**

Active management of the third stage of labour is widely used by both obstetricians and midwives in the UK. Syntometrine^® ^is usually used for vaginal births and oxytocin for caesarean births; when this is given and when the cord is clamped varies.

## Background

From birth of the baby to delivery of the placenta is known as the third stage of labour. These first few moments of their childs' life are an important time for the parents, but this is also a period during which some women are at risk of major haemorrhage. Haemorrhage following childbirth remains an important cause of maternal mortality, accounting for nine of the 14 deaths associated with haemorrhage in the latest UK confidential enquiry into maternal deaths [1]. In Africa and Asia postpartum haemorrhage accounts for about a third of maternal deaths [2].

Active management of the third stage of labour reduces the relative risk of postpartum haemorrhage (blood loss greater than 500 ml) by almost two thirds compared with physiological, or expectant, care [3]. Traditionally active management has three elements; use of a uterotonic drug, cord clamping, and controlled cord traction [3,4]. Routine use of a uterotonic drug more than halves the risk of postpartum haemorrhage [5], but the effects of timing of cord clamping [6] and controlled cord traction are unclear [7]. Optimum timing for cord clamping is particularly controversial, with the World Health Organisation [8], the International Confederation of Midwives [9] and the International Federation of Gynaecologists and Obstetricians [9] now recommending deferred cord clamping. Nevertheless, WHO describe this as 'weak recommendation, low quality evidence' [10]. In the UK, the National Institute for Health and Clinical Excellence (NICE) advises immediate cord clamping [11]. The lack of strong evidence to support some elements of active management, and conflicting recommendations and guidelines may contribute to variation in practice. To understand more about current practice for care during the third stage of labour in the UK, we conducted a postal survey of obstetricians and midwives.

## Methods

A questionnaire was posted to 2230 UK fellows and members of the Royal College of Gynaecologists and Obstetricians (RCOG) in August 2008. This excluded 1899 who had opted out of having their address used in this way. In September 2008 a similar questionnaire was sent to 2400 members of the Royal College of Midwives (RCM) in the UK. This was a 10% random sample, selected by computer from the RCM database of members. For both surveys, two postal reminders were sent. Responses were confidential and no data that might identify individuals were requested. Returned, completed questionnaires were considered indicative of consent to participate. Ethical approval is not required for a postal survey of current practice. The questionnaire was approved by both the RCOG and the RCM before they agreed to use of their mailing list.

Following a local pilot study [12] the questionnaires were modified and retested. Each questionnaire (see additional files 1 and 2) was on two sides of A4, and designed to be completed in three to four minutes. If respondents had not conducted or supervised births in the last twelve months they were asked to tick the appropriate box and return the questionnaire without answering any further questions. Questions asked about the use of active management and physiological care during the third stage for vaginal and caesarean births, and views about the need for more evidence from randomised trials to guide care during the third stage. Data were entered into Excel, and double checked for accuracy.

## Results

Overall, 53% (1194/2230) of the fellows and members of the RCOG responded of whom 926 (78%) had conducted or supervised births in the last 12 months; and 71% (1702/2400) of midwives responded of whom 1297 (76%) had conducted or supervised births in the last 12 months. Of the 1297 midwives, 792 worked in a consultant-led unit, 360 in the community, 180 in a midwife-led unit attached to a consultant-led unit and 110 in a stand alone midwife-led unit. These responses are not mutually exclusive, as some midwives practised in more than one setting.

Most obstetricians (93%) and midwives (73%) reported 'always or usually' using active management of the third stage of labour for vaginal births (Table 1). When using active management, two thirds of obstetricians and one third of midwives usually give the uterotonic drug with delivery of the anterior shoulder, and 94% of both groups 'always or usually' use controlled cord traction. For vaginal births Syntometrine^® ^(oxytocin and ergometrine) was the uterotonic usually used by 86% of midwives and 79% of obstetricians.

**Table 1:** Reported management of the third stage of labour for vaginal births

	Midwives n = 1297 (%)	Obstetricians n = 926 (%)
***How often do you use active management for the third stage?***		
always or usually	942 (73)	863 (93)
sometimes	291 (22)	60 (6)
rarely	60 (5)	1 (< 1)
never	4 (< 1)	-
no response	-	2 (< 1)
		
***When you use active management***		
*When is the prophylactic uterotonic drug usually given?*		
with delivery of the anterior shoulder	430 (33)	611 (66)
with delivery of the body	211(16)	162 (17)
after birth of the baby, before cord clamping	365 (28)	68 (7)
after birth of the baby, after cord clamping	261 (20)	72 (8)
timing varies	24 (2)	7 (1)
no response/never use active management	6 (< 1)	6 (1)
		
*Which prophylactic uterotonic drug is usually used?*		
intramuscular oxytocin	172 (13)	186 (20)
intramuscular syntometrine^®^	1118 (86)	728 (79)
other	1 (< 1)	9 (1)
no response/never use active management	6 (< 1)	3 (< 1)
		
*How often do you use controlled cord traction?*		
always or usually	1214 (94)	868 (94)
sometimes	57 (4)	49 (5)
rarely	19 (1)	6 (1)
never	3 (< 1)	3 (1)
do not use active management	4 (< 1)	-
		
***How often do you use physiological management for the third stage?***		
always or usually	121 (9)	21 (2)
sometimes	606 (47)	120 (13)
rarely	476 (37)	431 (47)
never	92 (7)	350 (38)
no response	2 (< 1)	4 (< 1)
		
***Usually record timing of cord clamping***	182 (14)	23 (3)

For active management of term births, 41% of midwives clamp the cord within 20 seconds compared to 74% of obstetricians (Table 2). For preterm births, 55% of midwives and 57% of obstetricians clamp within 20 seconds. Two thirds of midwives and half the obstetricians would not use physiological management for preterm births. For caesarean births, cord clamping is rarely later than 60 seconds (Table 3), and the uterotonic drug is usually given at or after birth of the baby. For 90% the uterotonic was intravenous oxytocin. Midwives and obstetricians definitions for early and late clamping are presented in Table 4.

**Table 2:** Reported timing of cord clamping for vaginal births

	Midwives n = 1297 (%)		Obstetricians n = 926 (%)	
	*term*	*preterm*	*term*	*preterm*
***When is the cord usually clamped?***				
*For active management*				
immediately or within 10 seconds	211 (17)	464 (36)	422 (46)	314 (34)
within 10 - 20 seconds	315 (24)	243 (19)	260 (28)	209 (23)
within 20 - 30 seconds	237 (18)	129 (10)	101 (11)	125 (13)
within 30 - 60 seconds	389 (30)	143 (11)	112 (12)	208 (22)
within 1-3 minutes	46 (4)	8 (1)	4 (< 1)	9 (1)
after 3 minutes	12 (1)	5 (< 1)	-	-
after cessation of pulsation	51 (4)	8 (1)	-	-
timing varies	27 (2)	17 (1)	9 (1)	15 (2)
do not conduct preterm births	-	215 (17)	-	-
no response/never use active management	9 (1)	60 (5)	18 (2)	46 (4)
				
***When is the cord usually clamped?***				
*For physiological management*				
within 1-30 seconds	21 (2)	71 (5)	79 (14)	68 (12)
within 31 - 60 seconds	24 (2)	28 (2)	42 (7)	44 (8)
within 1-3 minutes	28 (2)	4 (> 1)	21 (4)	17 (3)
after > 3 minutes	7 (1)	2 (> 1)	9 (2)	3 (1)
after cessation of pulsation	1050 (87)	132 (10)	231 (40)	97 (17)
timing varies	3	9 (1)	2 (1)	1 (< 1)
do not use physiological for this group	48 (4)	768 (59)	149 (26)	293 (51)
do not conduct preterm births	-	149 (11)	-	-
no response/never use physiological	116 (9)	134 (10)	392 (45)	403 (47)

**Table 3:** Reported management of the third stage of labour for Caesarean births

	Obstetricians n = 926 (%)	
*When is the prophylactic uterotonic drug usually given?*		
with delivery of the anterior shoulder	93	(10)
with delivery of the body	324	(35)
after birth of the baby, before cord clamping	203	(22)
after birth of the baby, after cord clamping	290	(31)
other*	9	(1)
no response	7	(< 1)
		
*Which prophylactic uterotonic drug is usually used?*		
intravenous oxytocin	837	(90)
intramuscular Syntometrine^®^	82	(9)
other**	2	(< 1)
no response	5	(< 1)
		
*For term births, when is the cord usually clamped?*		
immediately or within 10 seconds	509	(55)
within 10 - 20 seconds	227	(24)
within 20 - 30 seconds	80	(9)
within 30 - 60 seconds	77	(8)
after > 60 seconds	9	(1)
other^†^	3	(< 1)
no response	21	(2)
		
*For preterm births, when is the cord usually clamped?*		
immediately or within 10 seconds	396	(43)
within 10 - 20 seconds	180	(19)
within 20 - 30 seconds	127	(13)
within 30 - 60 seconds	150	(16)
after > 60 seconds	28	(3)
other^‡^	7	(1)
no response	38	(4)

*6 use intravenous oxytocin; 3 use both intramuscular oxytocin and Syntometrine^®^

**2 use intravenous infusion

^†^cord milked then clamped n = 1; after cessation of pulsation n = 2

^‡^depends on situation n = 5; cord milked then clamped n = 1; after cessation of pulsation n = 1

**Table 4:** Midwives and obstetricians definitions of early and late cord clamping

	Midwives n = 1297 (%)				Obstetricians n = 926 (%)			
	term		preterm		term		preterm	
***How would you define early clamping?***								
after 1-10 seconds	362	(28)	395	(30)	359	(39)	301	(33)
11-20 seconds	118	(9)	98	(8)	108	(12)	119	(13)
21-30 seconds	212	(16)	187	(14)	177	(17)	187	(20)
31-60 seconds	245	(19)	156	(12)	136	(19)	153	(17)
1-5 minutes	69	(5)	5	(< 1)	15	(2)	21	(2)
> 5 minutes	12	(1)	29	(2)	5	(< 1)	4	(< 1)
other	*34	(3)	**43	(3)	-	-	-	-
no response	245	(19)	384	(30)	126	(14)	141	(15)
								
***How would you define late clamping?***								
after 1-30 seconds	17	(1)	68	(5)	90	(10)	89	(10)
31-60 seconds	59	(5)	124	(10)	199	(21)	194	(21)
1-2 minutes	42	(3)	72	(6)	100	(11)	126	(14)
2-3 minutes	41	(3)	8	(< 1)	16	(2)	19	(2)
3-5 minutes	40	(3)	67	(5)	24	(2)	34	(4)
> 5 minutes	49	(4)	24	(2)	15	(2)	10	(1)
After cessation of pulsation	895	(69)	508	(39)	356	(38)	309	(33)
other	^†^6	(< 1)	^††^11	(1)	-	-	-	-
no response	148	(11)	415	(31)	126	(14)	145	(16)

* after cessation n = 1, after cessation of pulsation and variable n = 33

** before delivery of placenta n = 2, not applicable for preterm births n = 32, varies n = 9

^† ^varies n = 5, before delivery of placenta n = 1.

^†† ^varies n = 10, after delivery of placenta n = 1

Most midwives (89%) and obstetricians (81%) think more evidence from randomised trials is needed to guide care during the third stage (Table 5). The most popular question was when is best to clamp the cord.

**Table 5:** Midwives and obstetricians views on the need for more evidence to guide care during the third stage of labour

	Midwives n = 1297 (%)		Obstetricians n = 926 (%)	
*More evidence needed from randomised trials*	1151	(89)	747	(81)
no response	4	(<1)	19	(2)
				
*If more evidence needed, what do you think are the important questions?*				
when is best to clamp the cord	1032		655	
is placental drainage beneficial	913		505	
optimum timing for the prophylactic uterotonic drug	885		504	
is controlled cord traction beneficial	766		361	
other^‡^	98		32	

^‡ ^Midwives: management of retained placenta n = 8; water births n = 3; management of third stage n = 64; which uterotonic is best n = 22; cord milking n = 1

Obstetricians: which uterotonic n = 14; management of retained placenta n = 10; management of third stage n = 6; cord milking n = 2

## Discussion

Active management of the third stage of labour is widely used by both obstetricians and midwives in the UK. Nevertheless, our study suggests variation in how some elements are applied. The most widespread and uniform practice was controlled cord traction. Controlled cord traction has previously been reported to be widespread maternity unit policy in the UK and Ireland, but less common in other European countries [4,13]. In our survey obstetricians and midwives largely report using intramuscular Syntometrine^® ^(oxytocin plus ergometrine) as the prophylactic uterotonic drug for vaginal births. This is surprising as intramuscular oxytocin has a similar impact to Syntometrine^® ^on risk of postpartum haemorrhage, but with fewer adverse effects such as nausea, vomiting and hypertension [5]. Intramuscular oxytocin is also recommended by NICE [11]. Possible factors in the continued use of Syntometrine^® ^in the UK may be lack of awareness of the evidence and national guidelines, habit, or a belief that despite the evidence Syntometrine^® ^is associated with a lower risk of postpartum haemorrhage.

Timing of administration of the prophylactic uterotonic drug at vaginal birth varies. Giving the uterotonic drug with delivery of the anterior shoulder is usual policy for two thirds of UK maternity units [4]. Our survey suggests this policy is followed for vaginal births conducted by obstetricians, but midwives were more likely to give the uterotonic later. Similar practice for obstetricians and midwives has been reported elsewhere [14]. This may be because if the midwife is alone she will not be able to give the uterotonic drug until after the baby is born, whilst obstetricians are less likely to attend a delivery alone.

At caesarean section, most obstetricians report using intravenous oxytocin, as recommended by NICE [11]. Although we did not ask who was responsible for administering this, it would usually be the anaesthetist. Reported use of oxytocin at caesarean section is similar for anaesthetists and obstetricians, however [15]. Use of Syntometrine at caesarean section is surprising, as it is not recommended in the UK and is associated with more adverse effects [11].

When the cord is clamped also varied between midwives and obstetricians. Obstetricians largely clamp the cord within 60 seconds for both vaginal and caesarean births, which is similar to practice reported in Latin America, Africa and some European countries [4,13,16]. When asked to define early and late cord clamping, responses varied. This is not surprising, as there is no consensus about the definition of early and late clamping. The high level of no response to these questions probably also reflects uncertainty about the definitions. For term and preterm births obstetricians and midwives tended to give the same definitions of early and late clamping, the exception being that fewer midwives defined late clamping for preterm births as after cessation of pulsation.

Timing of cord clamping will influence placental transfusion as, if the cord is not clamped, blood flow usually continues for a few minutes. The additional blood volume transferred to the infant during this time is known as placental transfusion. For a term infant, placental transfusion gives the infant an additional median of 80-85 mls of blood [17-19], which contributes 23 ml per kilogramme, increasing blood volume at birth by 25%. Within a few hours the additional plasma from the placental transfusion is lost to the circulation, leaving a high red cell mass. This is quickly broken down and the iron stored. Restricting placental transfusion by immediate cord clamping may deprive the infant of 20-30 mg/kg of iron, sufficient for the needs of a newborn infant for around three months. Although there are few data, the relative reduction in blood volume and red cell mass following immediate clamping may be even greater for preterm infants than for those born at term, as a higher proportion of the intrauterine blood volume is sequestered in the placenta.

The terminology of 'early' and 'late' cord clamping is misleading. The Oxford English Dictionary defines early as 'before the due or usual time' and late as 'after the due or usual time' or 'after the proper time'. The implication is therefore that early clamping is the norm and good, and that late clamping is not the norm and bad. To avoid this implication in the language used for this topic, we suggest using immediate clamping and deferred clamping which is more neutral in tone.

Immediate cord clamping restricts placental transfusion. For term infants this leads to marginally less jaundice and less need for phototherapy at birth, and to lower iron stores in the first few months of life [6]. Data on many short term outcomes are incomplete, however, and there are no data on the childs' subsequent health and development [6]. Iron deficiency in the first few months of life is associated with neurodevelopmental delay, which may be irreversible [20,21]. Whether deferring cord clamping to allow placental transfusion improves neurodevelopment in early childhood is not known. Even a modest effect would have important public health implications, as anaemia and iron deficiency in early childhood remain common [21]. For preterm infants there is promising evidence that placental transfusion may have substantive benefits in the short term [22,23]. Again, there are no data on long term outcome. Large randomised trials are needed to evaluate timing of cord clamping and placental transfusion for both term and preterm births [24]. We are planning such trials, and would welcome collaboration with others planning similar studies.

The response rate from obstetricians was lower than expected, as our previous experience is that a 72% response is possible for a short postal questionnaire [25]. Nevertheless, this response rate is typical for surveys of doctors [26]. Factors in the low response may have been that the Royal College of Obstetricians and Gynaecologists has recently surveyed fellows and members to ask whether their contact details can be made available for external use; those who are not good at responding to surveys are therefore less likely to have opted out. This mailing list is also used regularly for surveys, which may decrease motivation to respond. Finally, those in training or temporary posts may not have kept their contact details up to date. A clinicians' current practice for care during the third stage is unlikely to have influenced their willingness to respond, however, and our data are therefore likely to be representative of obstetricians in the UK. The survey did not distinguish between practice within the NHS and the private sector.

We achieved a high response rate from midwives. Although our sample was from the Royal College of Midwives, it is likely that this is reasonably representative of UK practice overall. In the UK all practising midwives are registered with the Nursing and Midwifery Council, which currently has 35,305 midwives registered, compared to approximately 24 000 midwives who are members of the Royal College of Midwives. Our impression is that most practising midwives working within the NHS are members of the Royal College of Midwives. Midwives registered with the Nursing and Midwifery Council who are not members of the Royal College of Midwives may be either: not in clinical practice; in private practice, or members of the Association of Radical Midwives, a group which supports a physiological approach to childbirth whenever possible. The largest group is likely to be those who have maintained their registration, but are no longer in clinical practice. Nevertheless, it is possible that our survey under-reports the use of physiological care during the third stage.

For physiological care, we did not ask about the use of a prophylactic uterotonic drug or controlled cord traction. Our survey may therefore under report the use of these two interventions.

## Conclusions

Most obstetricians and midwives thought more evidence from randomised trials was needed to guide care in the third stage. The third stage of labour is a critical time, and generating reliable evidence to guide care should be a priority. This survey demonstrates that even when such evidence exists, it may not be implemented. Use of Syntometrine^® ^as the prophylactic uterotonic drug for vaginal births is a widespread habit amongst both midwives and obstetricians. It is time this habit was broken.

## Competing interests

The authors declare that they have no competing interests.

## Contributions of the authors

LD conceived the study. LD and DF drafted the protocol, with comments from DT. DF, RA and LD conducted the survey. The paper was drafted by DF and LD with comments from DT and RA. All authors read and approved the final manuscript.

## Pre-publication history

The pre-publication history for this paper can be accessed here:

http://www.biomedcentral.com/1471-2393/10/23/prepub

## Supplementary Material

Additional file 1 **Midwife's Questionnaire**. Questionnaire administered to Royal College of Midwives members asking about their current practice of the third stage of labour Click here for file

Additional file 2 **Obstetrician's Questionnaire**. Questionnaire administered to Royal College of Obstetricians and Gynaecologists members asking about their current practice of the third stage of labour Click here for file
