# Supplementary material for: Care during the third stage of labour: A postal survey of UK midwives and obstetricians
Source: BMC Pregnancy Childbirth. 2010 May 21;10:23. doi: 10.1186/1471-2393-10-23 (PMC2885994; doi:10.1186/1471-2393-10-23)
Supplement: Additional file 1 — Midwife's Questionnaire. Questionnaire administered to Royal College of Midwives members asking about their current practice of the third stage of labour [file 1471-2393-10-23-S1.DOC]

**Care during the third stage of labour**

This questionnaire asks about your own practice during the third stage, it should take no more than three or four minutes to complete. Thank you for your time.

*If you would like a copy of the survey results, please supply your email at the end of the questionnaire*

| **1. In the last twelve months have you conducted, or supervised, births?**  yes | | |  | | |  |
| --- | --- | --- | --- | --- | --- | --- |
| no | | |  | | |  |
| ***If no****, thank you for your time. Please return this questionnaire in the prepaid envelope provided* | | | | | |  |
| **2. In which year did you qualify as a midwife?** | | |  | | |  |
|  | | | | | |  |
| **3. Where do you usually undertake or supervise births?** community | | |  | | |  |
| *(tick one box only)* stand alone midwife led unit | | |  | | |  |
| midwife led unit attached to an obstetric unit | | |  | | |  |
| obstetric consultant led unit | | |  | | |  |
| other, please specify …………… .. … … … | | |  | | |  |
|  | | | | | |  |
| **4. How often do you use active management for the third stage?**  *tick one box only* | | | | | |  |
| always or usually | | |  | | |  |
| sometimes | | |  | | |  |
| rarely | | |  | | |  |
| never *(go to Q9)* | | |  | | |  |
|  | | | | | |  |
| **5. When using active management,** how soon after birth of the baby do you usually clamp the cord?  *tick one box for each column* | | | | | |  |
| ***term preterm*** | | | | | |  |
| immediately, or within 10 seconds |  |  |  | | |  |
| within 10 - 20 seconds |  |  |  | | |  |
| within 20 - 30 seconds |  |  |  | | |  |
| within 30 - 60 seconds |  |  |  | | |  |
| other, please specify ………………… |  | …………. |  | | |  |
|  | | | | | |  |
| **6. When using active management,** when do you usually give the prophylactic uterotonic drug?  *tick one box only* | | | | | |  |
| with delivery of the anterior shoulder | | |  | | |  |
| with delivery of the body | | |  | | |  |
| after birth of the baby, before cord clamping | | |  | | |  |
| after birth of the baby, after cord clamping | | |  | | |  |
| other, please specify ……………… … .. … | | |  | | |  |
|  | | | | | |  |
| **7. When using active management,** which prophylactic uterotonic drug do you usually use?  *tick one box* *only* | | | | | | |
| intramuscular oxytocin | | | | |  | |
| intramuscular syntometrine | | | | |  | |
| other, please specify ………………............. | | | |  | | |

| **8. When using active management,** how often do you use controlled cord traction? | | | | | | |
| --- | --- | --- | --- | --- | --- | --- |
| *(tick one box only)* always or usually | | | | | |  |
| sometimes | | | | | |  |
| rarely | | | | | |  |
| other, please specify ……..………………… | | | | | |  |
|  | | | | | |  |
| **9. How often do you use physiological management for the third stage?** | | | | | | |
| *(tick one box only) a*lways or usually | | | | |  | |
| sometimes | | | | |  | |
| rarely | | | | |  | |
| never *(go to Q12)* | | | | |  | |
|  | | | | | | |
| **10. When using physiological management for a *term* birth,** when do you usually clamp the cord? | | | | | | |
| time from birth of the baby to cord clamping | | | | Min | Sec | |
| ***or,*** if you clamp after cessation of cord pulsation, *tick here* | | | | |  | |
| ***or,*** if you do ***not*** use physiological management for a ***term birth,*** *tick here* | | | | |  | |
| **11. When using physiological management for a *preterm* birth,** when do you usually clamp the  cord? | | | | | | |
| time from birth of the baby to cord clamping | | | | Min | Sec | |
| **o*r,*** if you clamp after cessation of cord pulsation, *tick here* | | | | |  | |
| ***or,*** if you do ***not*** use physiological management for a ***preterm*** birth, *tick here* | | | | |  | |
| **12. How would you define *early* cord clamping for term and preterm births?** | | | | | | |
| ***term preterm*** | | | | | | |
| birth of the baby to cord clamping | Min | Sec |  | Min | Sec | |
|  | | | | | | |
| **13. How would you define *delayed or late* cord clamping for term and preterm births?** | | | | | | |
| ***term preterm*** | | | | | | |
| birth of the baby to cord clamping | Min | Sec |  | Min | Sec | |
| ***or****,* if after cessation of cord pulsation, *tick here* | |  |  | |  | |
|  | | | | | | |
| **14. Do you usually record the timing of cord clamping in the woman’s notes?** yes | | | | |  | |
| no | | | | |  | |
| **15. Do you think that there is a need for more research evidence from randomised trials to** | | | | | | |
| **guide care during the third stage?**  yes | | | | |  | |
| no | | | | |  | |
|  | | | | |  | |
| ***If yes***, what do you think are the important clinical questions? *tick all that apply* | | | | | | |
| optimum timing for the prophylactic uterotonic drug | | | | |  | |
| when is best to clamp the cord | | | | |  | |
| is controlled cord traction beneficial | | | | |  | |
| is placental drainage beneficial | | | | |  | |
| other, please specify..………………………………………………. | | | | |  | |

If you would like a copy of the survey results, please supply your email address:

………………………………………………………………………………………………………………………….

**Thank you for your help**

Please return completed questionnaires, in the prepaid envelope provided, to:

Diane Farrar, Senior Research Midwife

Obstetric Epidemiology, Bradford Institute for Health Research,

Temple Bank House, Bradford Royal Infirmary, Duckworth Lane, Bradford BD9 6RJ

Mobile: 07976745626; Email: diane.farrar@bradfordhospitals.nhs.uk
