# Supplementary material for: Care during the third stage of labour: A postal survey of UK midwives and obstetricians
Source: BMC Pregnancy Childbirth. 2010 May 21;10:23. doi: 10.1186/1471-2393-10-23 (PMC2885994; doi:10.1186/1471-2393-10-23)
Supplement: Additional file 2 — Obstetrician's Questionnaire. Questionnaire administered to Royal College of Obstetricians and Gynaecologists members asking about their current practice of the third stage of labour [file 1471-2393-10-23-S2.DOC]

**Care during the third stage of labour**

This questionnaire asks about current practice during the third stage, it should take no more than three or four minutes to complete. Thank you for your time.

*If you would like a copy of the survey results, please supply your email at the end of the questionnaire*

| **1. In which year did you obtain your MRCOG?** | | | | | | | | |  | | |
| --- | --- | --- | --- | --- | --- | --- | --- | --- | --- | --- | --- |
|  | | | | | | | | | | | |
| **2. In the last twelve months have you conducted, or supervised, any births?** yes | | | | | | | | |  | | |
| no | | | | | | | | |  | | |
| ***If no****, thank you for your time. Please return this questionnaire in the prepaid envelope provided*  **☞ Q3 - Q10 refer to vaginal births only** | | | | | | | | | | | |
|  |  | | | | | | | | | | |
| **3. How often do you use active management for the third stage?** always or usually | | | | | | | | | |  | |
| *(tick one box only)* sometimes | | | | | | | | | |  | |
| rarely | | | | | | | | | |  | |
| never (go to Q8) | | | | | | | | | |  | |
|  | | | | | | | | | |  | |
| **4. When you use active management,** when is the prophylactic uterotonic drug usually given? | | | | | | | | | | | |
| *(tick one box* *only)*  with delivery of the anterior shoulder | | | | | | | | | |  | |
| with delivery of the body | | | | | | | | | |  | |
| after birth of the baby, before cord clamping | | | | | | | | | |  | |
| after birth of the baby, after cord clamping | | | | | | | | | |  | |
| other, please specify… … … … … ............. | | | | | | | | | |  | |
|  | | | | | | | | | | | |
| **5. When you use active management,** which prophylactic uterotonic drug is usually used? | | | | | | | | | | | |
| *(tick one box* *only)*  intramuscular oxytocin | | | | | | | | | |  | |
| intramuscular syntometrine | | | | | | | | | |  | |
| other, please specify……………………. | | | | | | | | | |  | |
|  | | | | | | | | | | | |
| **6. When you use active management,** how long after birth is the cord usually clamped? | | | | | | | | | | | |
| *tick one box for each column* | | | | | | | | | | | |
| ***term*** ***preterm*** | | | | | | | | | | | |
| immediately or within 10 seconds | | | |  | |  | | | |  | |
| within 10 - 20 seconds | | | |  | |  | | | |  | |
| within 20 - 30 seconds | | | |  | |  | | | |  | |
| within 30 - 60 seconds | | | |  | |  | | | |  | |
| other, please specify ..……………… | | | |  | | ………….. | | | |  | |
|  | | | | | | | | | | | |
| **7. When you use active management,** how often do you use controlled cord traction? | | | | | | | | | | | |
| *(tick one box only)* always or usually | | | | | | | | | |  | |
| sometimes | | | | | | | | | |  | |
| rarely | | | | | | | | | |  | |
| other, please specify ………………......... | | | | | | | | | |  | |
|  | | | | | | | | | | | |
| **8. How often do you use physiological management for the third stage?** | | | | | | | | | | | |
| *(tick one box only)* always or usually | | | | | | | | |  | | |
| sometimes | | | | | | | | |  | | |
| rarely | | | | | | | | |  | | |
| never *(go to Q11)* | | | | | | | | |  | | |
|  | | | | | | | | | | | |
| **9. When you use physiological management for a *term* birth,** when is the cord usually clamped? | | | | | | | | | | | |
| time from birth of the baby to cord clamping | | | | | | | Min | | | Sec | |
| **o*r,*** if cord clamped after cessation of cord pulsation, *tick here* | | | | | | | | | |  | |
| ***or,*** if you do ***not*** use physiological management for a ***term birth,*** *tick here* | | | | | | | | | |  | |
| **10. When you use physiological management for a *preterm* birth,** when is the cord usually clamped? | | | | | | | | | | | |
| time from birth of the baby to cord clamping | | | | | | | Min | | | Sec | |
| ***or,*** if cord clamped after cessation of cord pulsation, *tick here* | | | | | | | | | |  | |
| ***or,*** if you do ***not*** use physiological management for a ***preterm*** birth, *tick here* | | | | | | | | | |  | |
| **☞ Q11 - Q13 refer to Caesarean births only** | | | | | | | | | |  | |
| **11. For a Caesarean birth, when is the prophylactic uterotonic drug usually given?** | | | | | | | | | | | |
| *(*tick *one* box only*)* with delivery of the anterior shoulder | | | | | | | | | |  | |
| with delivery of the body | | | | | | | | | |  | |
| after birth of the baby, before cord clamping | | | | | | | | | |  | |
| after birth of the baby, after cord clamping | | | | | | | | | |  | |
|  | | | | | | | | | | | |
| **12. For a Caesarean birth, which prophylactic uterotonic drug is usually used?** | | | | | | | | | | | |
| *(tick one box* *only)*  intravenous oxytocin | | | | | | | | | |  | |
| intramuscular syntometrine | | | | | | | | | |  | |
| other, please specify ……................................ | | | | | | | | | |  | |
|  | | | | | | | | | | | |
| **13. For a Caesarean birth, when is the cord usually clamped?** *tick one box for each column* | | | | | | | | | | | |
| ***term preterm*** | | | | | | | | | | | |
| immediately or within 10 seconds | | | | |  | | |  | |  | |
| within 10 - 20 seconds | | | | |  | | |  | |  | |
| within 20 - 30 seconds | | | | |  | | |  | |  | |
| within 30 - 60 seconds | | | | |  | | |  | |  | |
| after more than 1 minute | | | | |  | | |  | |  | |
| other, please specify …………………….. | | | | |  | | | ………... | |  | |
| **☞ Q14 - Q17 ask about your views** | | | | |  | | |  | |  | |
| **14. How would you define *early* cord clamping for term and preterm births?** | | | | | | | | | | | |
| ***term***  ***preterm*** | | | | | | | | | | | |
| birth of the baby to cord clamping | | Min | Sec | |  | | | Min | | Sec | |
|  | | | | | | | | | | | |
| **15. How would you define *delayed* or late, cord clamping for term and preterm births?** | | | | | | | | | | | |
| ***term***  ***preterm*** | | | | | | | | | | | |
| birth of the baby to cord clamping | | Min | Sec | |  | | | Min | | Sec | |
| ***or,*** if after cessation of cord pulsation, *tick here* | | |  | |  | | | | |  | |
|  | | | | | | | | | |  | |
| **16. Do you usually record the timing of cord clamping in the woman’s notes?** Yes | | | | | | | | | |  | |
| no | | | | | | | | | |  | |
| **17. Do you think we need more evidence from randomised trials to guide care during the third** | | | | | | | | | | | |
| **stage?**  yes | | | | | | | | | | |  |
| no | | | | | | | | | | |  |
| ***If yes***, what do you think are the important questions? | | | | | | | | | | |  |
| (*tick all that apply)*  optimum timing for the prophylactic uterotonic drug | | | | | | | | | | |  |
| when is best to clamp the cord | | | | | | | | | | |  |
| is controlled cord traction beneficial | | | | | | | | | | |  |
| is placental drainage beneficial | | | | | | | | | | |  |
| other, please specify…..…………………… | | | | | | | | | | |  |

If you would like a copy of the survey results, please supply your email address:

………………………………………………………………………………………………………………………….

**Thank you for your help**

Please return completed questionnaires in the prepaid envelope provided to:

Diane Farrar Senior Research Midwife

Obstetric Epidemiology, Bradford Institute for Health Research,

Temple Bank House, Bradford Royal Infirmary, Duckworth Lane, Bradford BD9 6RJ

Mobile: 07976745626; Email: diane.farrar@bradfordhospitals.nhs.uk
